# A case report and literature review: gastric cancer with Krukenberg tumor in a patient with situs inversus totalis

**DOI:** 10.3389/fonc.2025.1615123

**Published:** 2025-11-25

**Authors:** Xiaoli Jin, Jiankang Shen, Ru Zhou, Dongjie Shen, Jianming Yuan

**Affiliations:** Department of General Surgery, Ruijin Hospital Lu Wan Branch, Shanghai Jiaotong University, School of Medicine, Shanghai, China

**Keywords:** gastric cancer, Krukenberg tumor, situs inversus totalis, case report, literature review

## Abstract

The primary modes of metastasis of gastric cancer are through the bloodstream and lymph nodes. However, in cases such as the Krukenberg tumor (KT), gastric cancer cells can spread to organs within the abdominal cavity and develop implantation metastases. This report describes the treatment process for a patient with situs inversus totalis (SIT), gastric cancer, and a KT. A 63-year-old woman with SIT presented with abnormal vaginal bleeding and was diagnosed with gastric cancer with KT. After careful evaluation, the patient underwent total gastrectomy (Roux-en-Y esophagojejunostomy, antecolic, and D2 lymph node dissection), total hysterectomy plus double adnexectomy, and appendectomy. Considering the patient’s SIT, we devised a modified surgical strategy. The surgery was successful. Postoperative adjuvant chemotherapy was administered, and her current evaluation indicated stable disease. Although gastric cancer complicated by KT is rare, it occurs in some cases. Surgical treatment should be considered if both the primary and secondary lesions can be radically resected and supplemented with systemic chemotherapy. For patients with SIT, the surgical approach should be appropriately adjusted with careful preoperative evaluation to ensure procedural safety.

## 1 Introduction

Gastric cancer is a highly prevalent malignancy of the digestive tract that ranks fifth in terms of morbidity (5.6%) and mortality (7.7%) (1). The primary modes of metastasis of gastric cancer are through the bloodstream and lymph nodes. However, in some cases, gastric cancer cells can spread to organs within the abdominal cavity and develop implantation metastases. One well-known example is the Krukenberg tumor (KT), which refers to ovarian implantation metastasis. KT was named after Dr. Friedrich Ernst Krukenberg to describe a type of ovarian metastatic tumor. It usually refers to a metastatic signet ring cell adenocarcinoma of the ovary characterized by mucin-rich signet ring cells (2). The primary site of KT is 70% likely to be the stomach, but it can also originate from organs such as the colon, appendix, breast, and biliary tract (3). The incidence of gastric cancer with KT in females is approximately 5–10%. The proportion of premenopausal women affected by this condition is higher than that of postmenopausal women (4). Situs inversus totalis (SIT) is a rare congenital structural anomaly that falls under the category of laterality disorders. It occurs during early development and is regulated by a complex interplay between signaling molecules and genes. SIT is characterized by a complete mirror reversal of the abdominal and thoracic organs. The incidence of SIT is approximately 1 in 10,000 individuals, with a slightly higher incidence in men (1.5:1 ratio) (5). Patients with SIT often exhibit atypical clinical manifestations that can complicate the diagnostic process. In addition, their unique anatomical structures pose significant challenges for surgeons. We recently treated a patient with SIT, gastric cancer, and KT at our hospital. The remainder of this paper is organized as follows.

## 2 Case description

**Figure 1:**
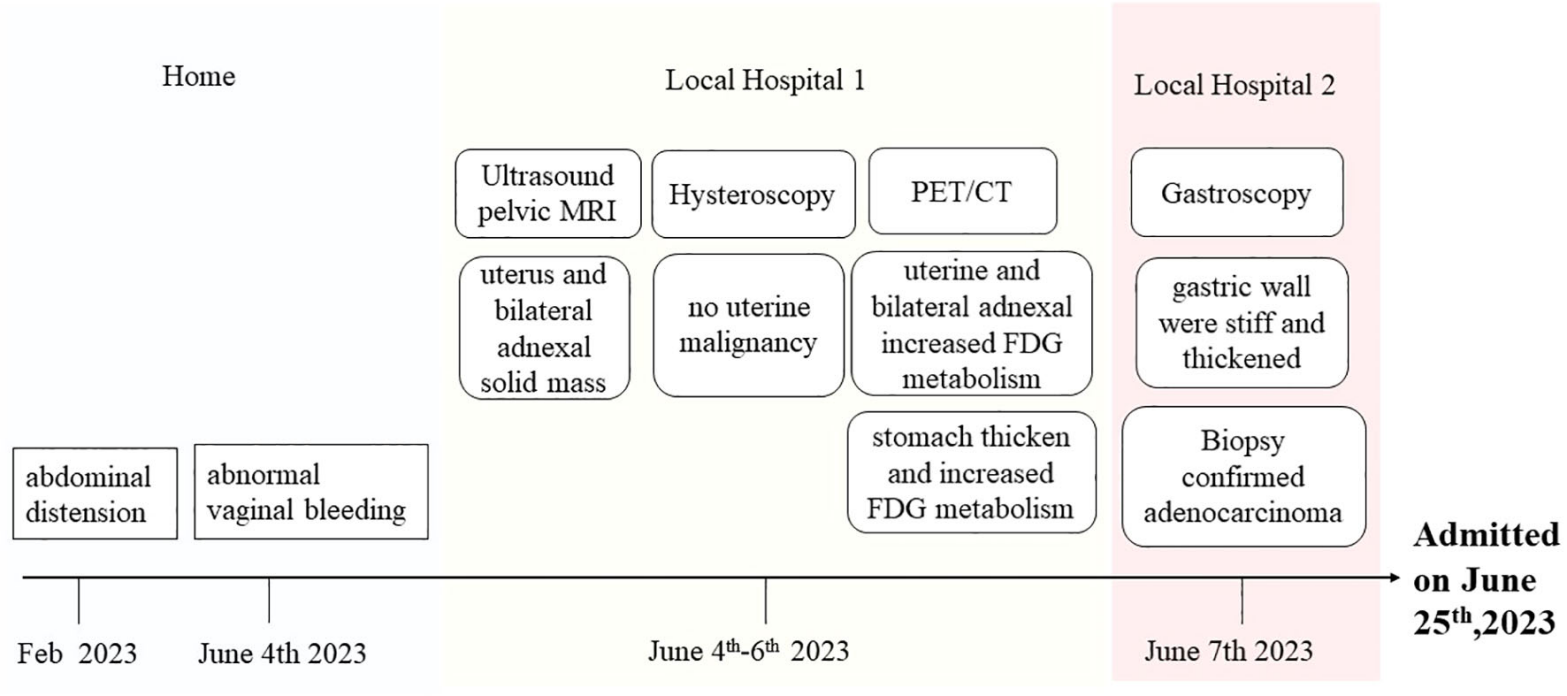
Patient’s complete medical history.

A 63-year-old woman was admitted to our hospital on June 25^th^, 2023, with the discovery of a malignant tumor in her stomach. In a review of the systems, she was found to be positive for abdominal distension after meals that persisted for 4 months, and experienced abnormal vaginal bleeding for 3 weeks, which prompted her to visit a local hospital. She had previously undergone a laparoscopic cholecystectomy at Ruijin Hospital for cholecystolithiasis treatment. In addition, she had no history of chronic diseases, surgeries, or alcohol or tobacco abuse. She gave birth once and had no history of miscarriage. She reached menopause at the age of 50 years and denied any personal or family history of cancer. No abnormalities were found during physical examination upon admission. Outpatient ultrasound and pelvic MRI at a local hospital revealed the presence of a solid mass in the uterus and bilateral adnexal areas. The medical team considered the possibility of bilateral primary malignant ovarian tumors or ovarian metastatic carcinoma. However, upon performing a hysteroscopy, no signs of uterine malignancy were revealed. Then she underwent a positron emission tomography/computed tomography (PET/CT) examination, which revealed a rare mirror inversion of the chest and abdominal organs. According to PET/CT examination reports, abnormally increased fluorodeoxyglucose (FDG) metabolism was observed in the uterine cavity and bilateral adnexal soft tissue. Given that the lesion affected both the adnexa and uterus, endometrial tumors, such as ovarian endometrioid malignant tumors and endometrial malignant tumors, were considered the most likely possibilities. Furthermore, examination revealed significant thickening of the stomach mucosa in the gastric body and antrum, including the greater and lesser curvatures, with abnormally increased FDG metabolism. This finding suggests the possibility of hypertrophic gastritis and malignant tumors (lymphoma or gastric cancer). No lesions were found during the previous hysteroscopy; hence, the patient was transferred to another hospital for gastroscopy. The results revealed that the gastric body and walls were stiff and thick, respectively. This condition involved the gastric angle and antrum. Biopsy confirmed the presence of a poorly differentiated adenocarcinoma, with some sections showing signet ring cell carcinoma. The patient was admitted to our hospital for surgery. The patient’s medical history during the visit is shown in **Figure 1**.

**Figure 2:**
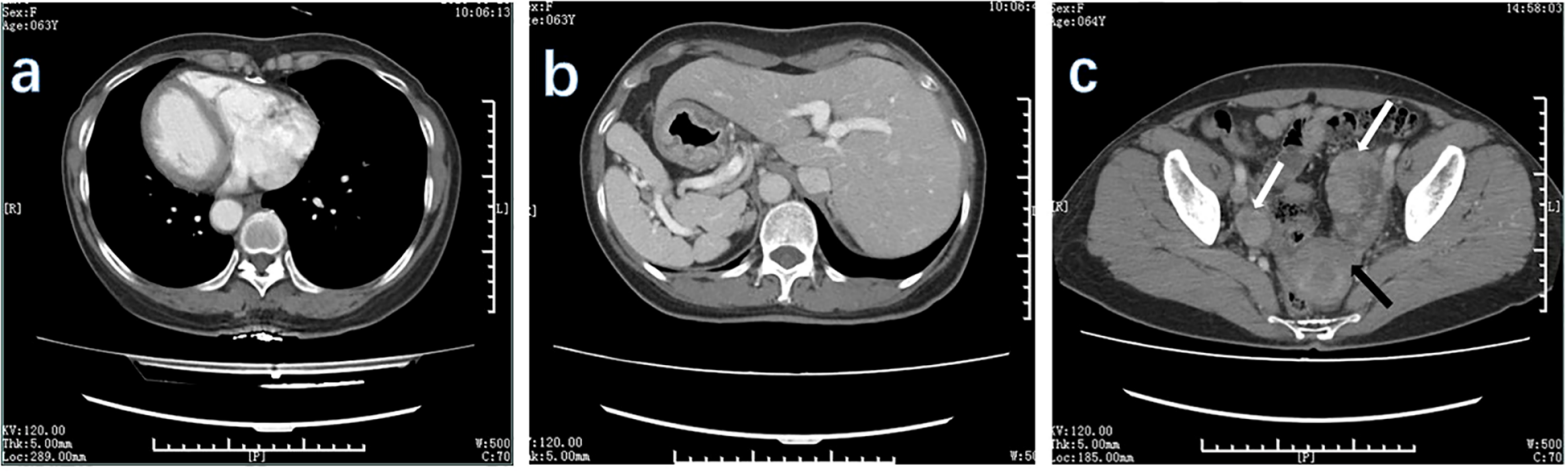
CT scan of the patient. The CT scan of this patient showed mirror inversion of the organs. **(A)** The patient’s chest organs were mirror-inverted. **(B)** The patient’s abdominal organs are mirror-inverted, and the gastric wall is thickened. **(C)** The patient’s pelvic organs show mirror inversion. White arrows show lesions in the adnexal region, and black arrows show lesions in the uterus.

Preoperative examination revealed a slightly poor nutritional status, with an albumin level of 34 g/L. No evidence of anemia. The tumor marker levels were mostly within the normal range, except for a slightly elevated carbohydrate antigen 72–4 level of 53.66 U/ml. Enhanced CT examinations of the chest and entire abdomen were conducted after admission to visually assess the visceral structure and lesions of the patient. This confirmed the diagnosis of SIT (**Figure 2**). The gastric wall was thickened, and bilateral soft tissue-occupying lesions in the adnexal region were observed. The preliminary diagnosis was gastric cancer with bilateral ovarian metastases (KT).

The patient underwent surgery on the 4^th^ hospital day. Initially, laparoscopic exploration was performed through the scar of the subumbilical puncture hole, and no widespread miliary metastasis was observed in the abdominal cavity. Subsequently, open abdominal surgery was performed. During surgery, all internal organs were returned to their normal positions. Adhesions were observed in the left upper abdomen, including in the liver, duodenum, greater omentum, and gallbladder fossa. The entire stomach was rigid and leather-like. Multiple enlarged lymph nodes with a maximum diameter of 2 cm were found around the stomach. Large hard masses were discovered bilaterally in the adnexa, measuring 8 × 6 × 5 cm on the left side and 5 × 4 × 4 cm on the right side.

During an on-stage consultation with gynecologists, meticulous examination of the pelvic tissue was conducted. Multiple myoma-like protrusions were observed in the uterus. A 6 × 6 cm solid-cystic mass with an intact capsule and firm texture was found in the left ovary. It exhibited mobility. Additionally, two masses measuring 3 × 3 cm and 2 × 2 cm with intact capsules, firm texture, and mobility were found in the right ovary. The appendix adhered to the right ovary. The fallopian tubes appeared normal, whereas significant edema was observed in the uterus and adnexa. After evaluation, both the gastric and ovarian lesions were deemed suitable for radical resection. In collaboration with a gynecologist, we performed a comprehensive procedure involving total gastrectomy (Roux-en-Y esophagojejunostomy, antecolic, and D2 lymph node dissection), total hysterectomy with double adnexectomy, and appendectomy. The abdominal surgery took 220 min, with a blood loss of approximately 150 ml. The pelvic surgery lasted approximately 100 min, and blood loss was approximately 200 ml. Fortunately, no complications occurred during surgery.

In conventional total gastrectomy, after opening the gastrocolic ligament, we typically proceed to sequentially divide the perigastric vessels in the following order: 1. It begins at the greater curvature of the antrum and proceeds along the greater curvature of the gastric body until it reaches the fundus of the stomach (right gastroepiploic vessels, left gastroepiploic vessels, and short gastric vessels). 2. We returned to the lesser curvature of the antrum (left gastric artery) and addressed the pylorus. 3. The cardia was then cut along the lesser curvature of the gastric body (right gastric artery). The esophagus was the final part to be removed.

**Figure 3:**
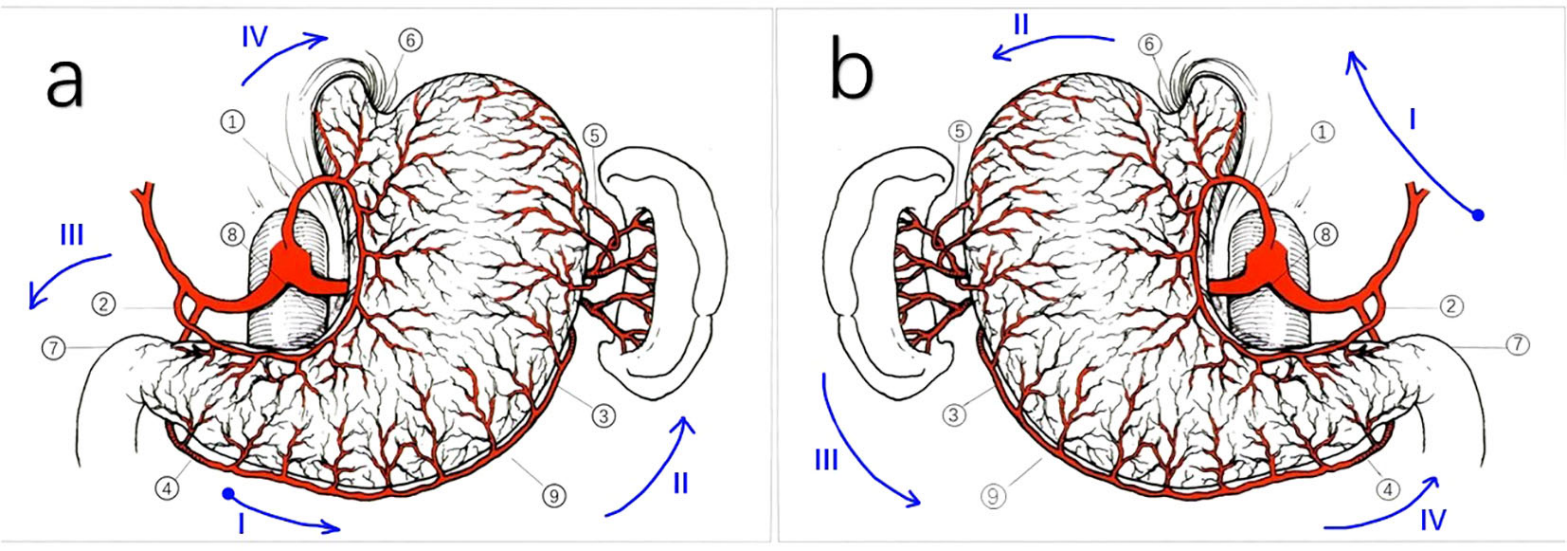
Schematic representation of the perigastric vessels in normal patients and patients with SIT. Schematic representation of the perigastric vessels in patients with SIT is a mirror inversion of. **(A)** Schematic representation of the perigastric vessels in normal patients. (1) Left gastric artery; (2) Right gastric artery; (3) Left gastroepiploic artery; (4) Right gastroepiploic artery; (5) Short gastric arteries; (6) Cardia; (7) Pylorus; (8) The lesser curvature; (9) The greater curvature. *: order in conventional surgery: (4) (3) (5) (2) (7) (1) (6). **(B)** Schematic representation of the perigastric vessels in our SIT case. (1) Left gastric artery; (2) Right gastric artery; (3) Left gastroepiploic artery; (4) Right gastroepiploic artery; (5) Short gastric arteries; (6) Cardia; (7) Pylorus; (8) The lesser curvature;(9)The greater curvature. *: order in our case: (2) (1) (6) (5) (3) (4) (7). The blue arrows in both **(a, b)** indicate the path of the surgical operation, with the blue dots representing the starting position. Roman letters represent surgical steps.

Considering the patient’s SIT condition, we modified the surgical approach after opening the gastrocolic ligament as follows: 1. Starting from the lesser curvature of the antrum; 2. After progressing counterclockwise from the lesser curvature of the gastric body to the cardia, fundus, greater curvature of the gastric body, and greater curvature of the antrum, the pylorus was finally excised. 3. The esophagus was severed, and the short gastric and left gastroepiploic vessels were addressed (**Figure 3**). This modification facilitates tissue traction and lymph node clearance when removing short gastric vessels.

**Figure 4:**
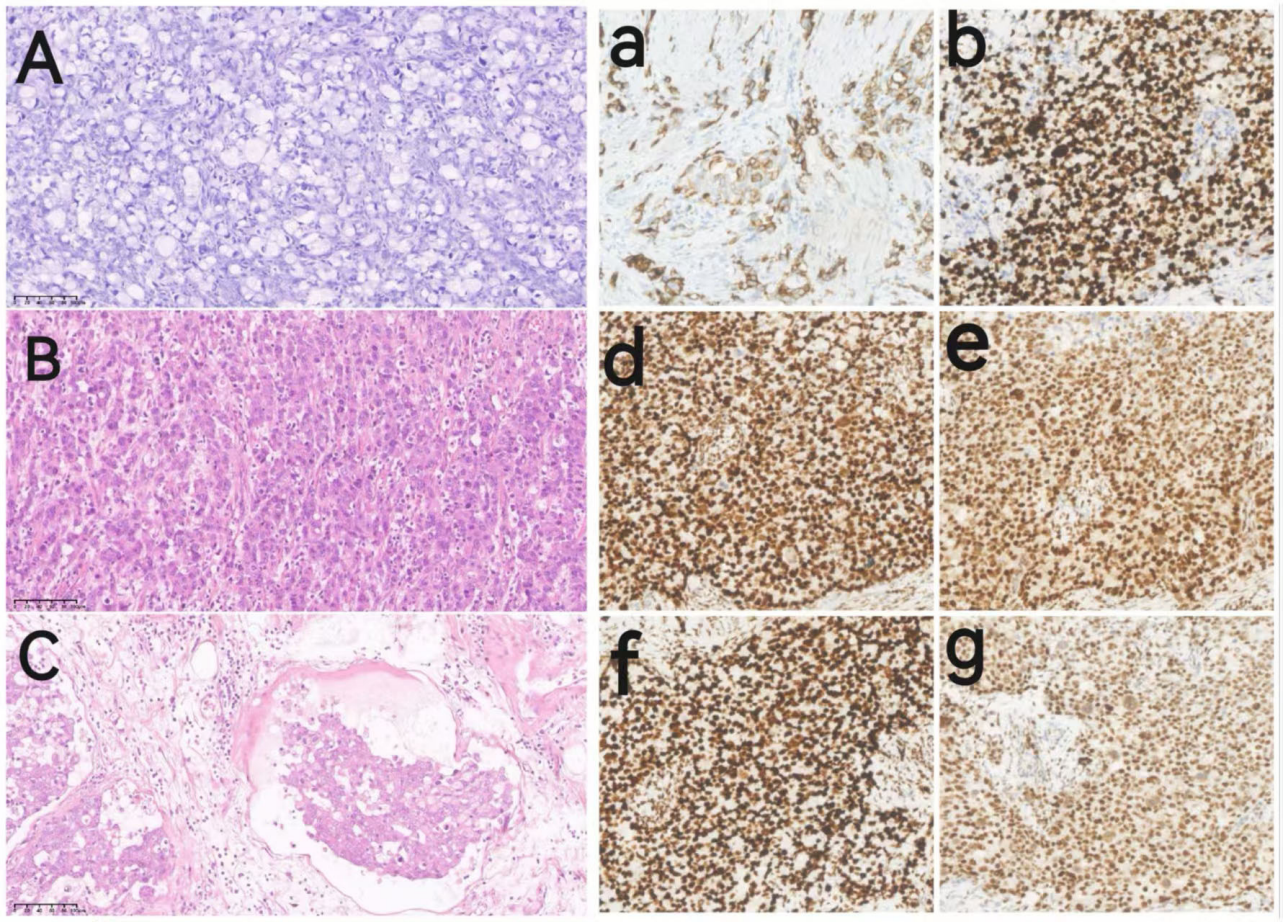
Pathological images of the patient. Images **(A–C)** show hematoxylin-eosin (HE) staining images of the pathological tissues(20^9^magnification); Images **(a, b, d–g)** show immunohistochemical (IHC) images of the pathological tissues (20^9^magnification): **(A)** HE staining for ovarian metastatic signet-ring cell carcinoma. **(B)** HE staining for gastric adenocarcinoma. **(C)** HE staining of gastric adenocarcinoma with intravascular tumor thrombus and visible signet ring cells. **(a)** IHC of CK7; **(b)** IHC of Ki-67; **(d)** IHC of MLH1; **(e)** IHC of MSH2; **(f)** IHC of MSH6; **(g)** IHC of PMS2.

The gastric specimen revealed a tumor measuring approximately 9 × 7 × 1 cm, located in the greater curvature of the gastric body and antrum. It was classified as a diffusely infiltrating tumor (Borrmann type IV). Paraffin pathology revealed a poorly differentiated adenocarcinoma with partial signet ring cell carcinoma penetrating the serosa (T4a). A tumor thrombus was present in the blood vessels, and local nerve invasion was observed. No metastasis or tumor invasion was detected in the omentum or bilateral resection margins. Overall, 33 out of the 45 perigastric lymph nodes had cancer metastasis (N3). Poorly differentiated adenocarcinoma tissues were also discovered in both ovaries, with involvement of the bilateral fallopian tubes and appendices. This finding was consistent with metastasis from gastric adenocarcinoma (KT), as supported by the patient’s history and immunohistochemical results. Multiple leiomyomas were identified in the uterus; however, no carcinomas were observed. Immunohistochemical results were as follows: HER2 (0), AE1/AE3 (+), CK7 (+), MLH1 (protein expression), PMS2 (protein expression), MSH2 (protein expression), MSH6 (protein expression), and Ki67 (80%+). Pathological images are shown in **Figure 4**. Based on these findings, the patient was staged with T4N3M1.

**Figure 5:**
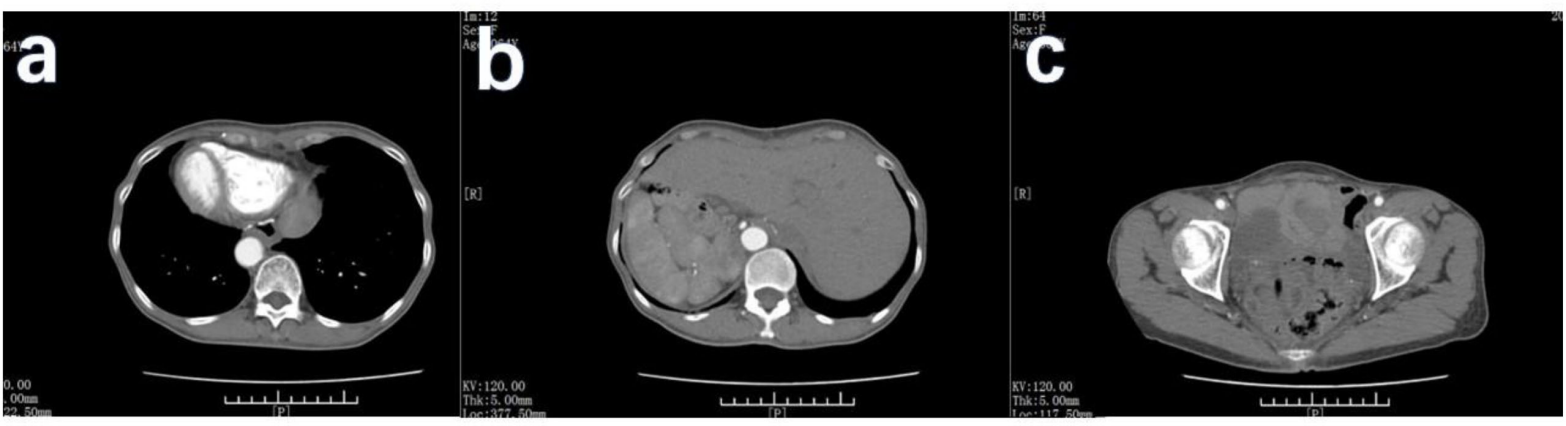
CT scan of the patient 11 months after the operation. **(A)** No new tumors were detected in the patient’s anastomotic area. **(B)** The patient’s liver was in mirror inversion, and no metastasis was found. **(C)** No new lesions in the patient’s pelvic area.

The patient was safely returned to the ward after surgery and began receiving total parenteral nutrition. Once the patient resumed flatus, enteral nutrition was gradually introduced. The patient started intraperitoneal chemotherapy on the 14^th^ postoperative day with the following regimens: cisplatinum 40 mg intraperitoneal instillation D1-D3. After completing the D2 chemotherapy, the patient experienced noticeable abdominal distension, nausea, and loss of appetite. These symptoms made it difficult for her to tolerate further treatment, leading to the discontinuation of intraperitoneal chemotherapy. The patient was discharged on the 22^nd^ postoperative day when her condition improved. After a short period of recuperation, the patient was referred to the oncology department for continued treatment. On August 9, 2023, according to the recommendations of the Chinese Society of Clinical Oncology guidelines, she received nine rounds of immunotherapy (sintilimab) along with chemotherapy (oxaliplatin + Teysuno [S-1], also called the SOX regimen). Due to the patient’s significant adverse reactions, including severe nausea and vomiting, the doses of both oxaliplatin and S-1 were reduced to 85% of the original dose from the second treatment onwards. From February 5, 2024, the patient received five cycles of sintilimab plus Teysuno therapy. The patient underwent abdominal CT on May 21, 2024, before the sixth immunotherapy session, which showed no new tumors in the anastomosis, liver, or pelvic area (**Figure 5**). Evaluation of the effectiveness of treatment indicated stable disease. She was transferred to a local hospital for continued treatment starting in June 2024. Owing to the lack of data sharing, the current condition of the patient could only be learned through telephone follow-ups and was found to be stable.

## 3 Discussion

Gastric cancer is a malignant tumor of the digestive tract. The primary mode of metastasis is through the bloodstream or lymph nodes, whereas abdominal cavity implantation is uncommon. In Asia, gastric cancer is the primary site for KT (3). A study involving 155 women with unresectable advanced or recurrent gastric cancer revealed that 9.7% had ovarian metastases and were younger than those without metastasis (6). SIT is a rare congenital structural anomaly. A systematic analysis (7) revealed that the highest number of patients with cancer and SIT was found in Asia, particularly in Japan, China, and South Korea, with Japan having the highest prevalence. Among the patients with SIT, the most frequently observed cancers are those affecting the stomach. This may be attributed to the high incidence of gastric cancer in Japan. However, there is currently no evidence confirming a direct relationship between SIT and malignant tumors.

### 3.1 Surgical indications for synchronous gastric and ovarian metastases

Uncertainties still exist regarding the treatment of patients with gastric cancer with KT. It is commonly accepted that Surgery is not the preferred option in cases where distant metastasis is present. Surgical intervention is typically reserved for patients with gastric cancer experiencing complications such as hemorrhage, perforation, or obstruction. However, it is important to note that in such cases, surgery is considered palliative rather than curative. However, the indications and extent of surgery in patients with synchronous gastric cancer and ovarian metastasis remain controversial. Currently, most studies suggest that if the primary lesion is resectable, removing the metastatic tumor may improve prognosis. Ovarian metastasectomy provides survival benefits in both synchronous and metachronous cases (8). In cases where gastric cancer and metastatic organs are deemed suitable for complete resection, excluding obvious ascites, extensive peritoneal implantation, and distant metastasis in important organs, the survival time of patients can be prolonged by surgically removing both the primary tumor and ovarian metastasis (9). Survival analysis also confirmed that in cases of synchronous gastric cancer complicated by KT, surgery should be actively performed if the lesion can be completely removed (10). However, in a study conducted by Hao et al. (11), prolonged survival was not observed in patients who underwent palliative resection for KT and gastric cancer. Therefore, a comprehensive treatment approach involving a multidisciplinary assessment is preferable. One study demonstrated that patients who underwent radical gastrectomy, ovarian metastasectomy, and chemotherapy had a more favorable prognosis (4).

Hyperthermic intraperitoneal chemotherapy (HIPEC) is a popular research topic for the treatment of advanced gastric cancer, particularly in patients with peritoneal metastases. HIPEC is used to prevent and treat peritoneal cancer and malignant ascites by heating the perfusion solution containing chemotherapy drugs to the therapeutic temperature and perfusing it into the abdominal cavity of patients for a certain period. Many studies have confirmed the effectiveness and safety of HIPEC combined with cytoreductive surgery in the treatment of patients with peritoneal carcinomatosis from malignant tumors, such as gastric, ovarian, and colorectal cancers (12, 13). As peritoneal metastasis of malignant tumors indicates poor prognosis and treatment effects, it is particularly important to prevent peritoneal recurrence in high-risk patients after radical surgery. Prophylactic HIPEC combined with radical D2 gastrectomy can result in favorable survival and peritoneal recurrence rates in locally advanced gastric cancer, with acceptable morbidity (14). Therefore, adjuvant intraperitoneal chemotherapy should be administered immediately after radical resection in patients who are considered to be at high risk of peritoneal recurrence (15). Although there are still controversies regarding the mode, temperature, duration, and dose of medication, first-line drugs for HIPEC mainly include mitomycin, platinum, and 5-Fu (15, 16).

In this particular case, the patient was diagnosed with synchronous gastric cancer and KT, and no ascites or peritoneal metastasis was detected during the preoperative evaluation. Consequently, radical surgery was conducted simultaneously for both gastric and ovarian tumors. Prophylactic intraperitoneal chemotherapy was administered postoperatively. Conventional intraperitoneal chemotherapy rather than HIPEC was used because of equipment limitations. Unfortunately, the patient could not tolerate the treatment; therefore, only systemic chemotherapy was administered. The patient remained in a stable condition for more than a year following surgery. Thus far, the therapeutic outcomes appear to be satisfactory. No evidence of tumor recurrence or metastasis was observed.

### 3.2 Surgical approach for patients with SIT and gastric cancer

In addition to the rare condition of gastric cancer with KT, the patient also had a rare condition known as SIT. Owing to the opposite anatomical structure in patients with SIT, identifying and managing anatomical structures during surgery becomes more challenging than in ordinary patients. Furthermore, experienced surgeons often develop a set of standardized surgical procedures through years of clinical practice, enabling them to perform operations successfully. However, when encountering a patient whose anatomical structure is completely reversed, particularly in cases of asymmetric organs, the surgeon’s previous experience and established procedures are challenged. Surgeons worldwide are actively seeking methods to overcome these challenges. Strategies, such as identifying key anatomical landmarks or fusing fascial spaces and utilizing microsurgical planes to locate vessels, are considered to significantly enhance the success rate of surgeries (17).

Most of the literature on surgeries for patients with SIT is in the form of case reports, owing to its rarity. We conducted a search on the PubMed database using the keywords “total gastrectomy” and “SIT” from 2014 to 2024. After excluding non-English and non-full-text articles, only eight cases were found. When the keyword “KT” was added, the search yielded zero results. Consequently, we compiled a summary of these eight cases, along with the current case, in **Table 1** (18–25).

**Table 1:** Summary of reported cases of SIT and total gastrectomy in the literature.

Study	Liu hong bo (18)	Young Lee (19)	Toshiaki (20)	Kengo Shibata (21)	Mamoru Morimoto (22)	Tsutomu Namikawa (23)	Yinghao Cao (24)	Taro Isobe (25)	Jin Xiaoli
Nationality	China	South Korea	Japan	Japan	Japan	Japan	China	Japan	China
Year of publication	2023	2023	2021	2018	2015	2018	2017	2015	2023
Age	55	79	84	79	58	66	60	79	63
Gender	M	F	M	M	M	F	M	F	F
Adjustments in surgical approach	opposite to the conventional	use special equipment	opposite to the conventional	modify the punching position	modify the punching position	N/M	modify the surgical approach	preoperative visceral 3D reconstruction	modify the surgical approach
Tumor Location	gastric cardia	posterior of the gastric body	the U region	posterior of the gastric body	posterior side, lesser curvature, gastric body	lesser curvature, gastric body	lesser curvature, gastroesophageal junction	anterior side, lesser curvature, the body to the angular incisure	greater curvature, gastric body and antrum
Maximum tumor diameter	2.5cm	4cm	N/M	N/M	2.5cm	12cm	1.4cm	N/M	9cm
Stage	pT3N3aM0	pT4aN0M0	pT3N2M0	pT3N1M0	pT1aN0M0	pT1N0M0	pT3N1M0	pT4aN3bM0	pT4aN3M1
Surgical technique	L-total gastrectomy	L- total gastrectomy(single-port)	R-total gastrectomy	L-total gastrectomy	L-total gastrectomy	total gastrectomy	R-total gastrectomy	total gastrectomy	total gastrectomy
Anastomosis method	RY	RY	intracorporal esophago jejunostomy by circular method	extracorporeal RY and intracorporeal overlap method of esophagojejunostomy	overlap method of esophagojejunostomy	RY	hand-sewn RY jejunoesophageal anastomosis	N/M	RY
Extent of resection	standard D2 LND	D1+ LND	D2 LND	modified D2 LND (without splenectomy)	D1+ dissection	regional LND	D2 LND	D2 LND (including splenectomy)	D2 LND (without splenectomy)
Surgical duration	168min	269min	N/M	232 min	359min	375min	N/M	288min	220min
Amount of bleeding	50mL	N/M	30mL	110 mL	90mL	380mL	N/M	150mL	150mL
Complication	no	no	no	no	no	no	no	no	no

LND, lymph node dissection; RY, Roux-en-Y reconstruction; L-total gastrectomy, laparoscopy-assisted total gastrectomy; R-total gastrectomy, robot-assisted total gastrectomy; N/M, not mentioned.

All cases were from Asia, with Japan being the majority (**Table 1**). Most patients had advanced gastric cancer, with only one case in the early stages. The main surgical approaches employed were laparoscopic or robot-assisted surgery, with only two patients (including the present case) undergoing open surgery. Nearly all reports mentioned preoperative or intraoperative adjustments to the surgical plan for patients with SIT, including adjustment of the position of the puncture hole, addition of special instruments, and preoperative 3D vascular reconstruction. As the literature consists of a limited number of case reports, descriptions of patient prognosis and outcomes mostly focus on surgical safety and the patient’s condition before the submission of the case. However, data on long-term prognosis and outcomes are lacking. A more comprehensive data analysis is needed to verify the long-term prognosis of patients with SIT undergoing total gastrectomy.

During the treatment of this patient, it was observed that while surgery for patients with SIT presented additional challenges, surgeons were able to adapt the sequence of resection or the surgical approach of total gastrectomy. These adjustments aimed to address the unconventional aspects of the surgery, ultimately making it more manageable for patients with SIT.

Compared with the conventional surgical approach, our operation involved significant changes. Instead of treating the vessels of the greater curvature from the antrum to the gastric body until the fundus and cutting off the esophagus, we modified the sequence as follows: 1. Vessels in the greater curvature of the stomach were treated from the gastric body to the antrum. 2. Esophageal dissection was performed as the initial step, followed by the dissection of the short stomach and left gastroepiploic vessels.

Most surgeons are right-handed; therefore, conventional surgical methods are typically designed for right-hand operations with left-hand assistance. However, when dealing with the left gastric vessels and short gastric vessels in patients with SIT using the conventional approach, there are several difficulties. If the operation is performed from the front of the stomach, the arms would cross over each other, making the procedure challenging. However, if the operation is performed from the rear of the stomach, the view is limited to the entire gastric body. In treating this patient, we adopted a modified approach, in which we first cut off the esophagus. This allowed us to pull the gastric body downward, providing better exposure of the operating area to the short gastric vessels. Then we treated the vessels in the greater curvature of the stomach in a clockwise direction. This approach aims to restore the conventional surgical methods of right-hand operation with left-hand assistance, thereby avoiding the interference caused by crossed arms and ensuring safe operation.

## 4 Conclusion

In conclusion, although gastric cancer complicated by KT is rare, it occurs in some cases. When both primary and secondary lesions can be radically resected, surgical treatment should be considered first and supplemented with systemic chemotherapy. For patients with SIT, the anatomy should be carefully evaluated before surgery, and the surgical approach should be adjusted appropriately during the operation to ensure the safety of the procedure.

## Data Availability

The raw data supporting the conclusions of this article will be made available by the authors, without undue reservation.
